# Next-generation fungal identification using target enrichment and Nanopore sequencing

**DOI:** 10.1186/s12864-023-09691-w

**Published:** 2023-10-02

**Authors:** Pei-Ling Yu, James C. Fulton, Owen H. Hudson, Jose C. Huguet-Tapia, Jeremy T. Brawner

**Affiliations:** 1 Department of Plant Pathology, University of Florida, Gainesville, FL 32611 USA; 2 Florida Department of Agriculture and Consumer Services, Division of Plant Industry, Gainesville, FL 32608 USA

**Keywords:** Oxford Nanopore Technologies, Probe-based target sequencing, Fungal identification

## Abstract

**Background:**

Rapid and accurate pathogen identification is required for disease management. Compared to sequencing entire genomes, targeted sequencing may be used to direct sequencing resources to genes of interest for microbe identification and mitigate the low resolution that single-locus molecular identification provides. This work describes a broad-spectrum fungal identification tool developed to focus high-throughput Nanopore sequencing on genes commonly employed for disease diagnostics and phylogenetic inference.

**Results:**

Orthologs of targeted genes were extracted from 386 reference genomes of fungal species spanning six phyla to identify homologous regions that were used to design the baits used for enrichment. To reduce the cost of producing probes without diminishing the phylogenetic power, DNA sequences were first clustered, and then consensus sequences within each cluster were identified to produce 26,000 probes that targeted 114 genes. To test the efficacy of our probes, we applied the technique to three species representing Ascomycota and Basidiomycota fungi. The efficiency of enrichment, quantified as mean target coverage over the mean genome-wide coverage, ranged from 200 to 300. Furthermore, enrichment of long reads increased the depth of coverage across the targeted genes and into non-coding flanking sequence. The assemblies generated from enriched samples provided well-resolved phylogenetic trees for taxonomic assignment and molecular identification.

**Conclusions:**

Our work provides data to support the utility of targeted Nanopore sequencing for fungal identification and provides a platform that may be extended for use with other phytopathogens.

**Supplementary Information:**

The online version contains supplementary material available at 10.1186/s12864-023-09691-w.

## Background

Characterizing fungi is challenging given their diversity [1, 2], similarities in morphological features [3, 4], and the frequent difficulties that arise when culturing fungal isolates. Due to these challenges, molecular-based identification techniques are widely used by mycologists and plant disease diagnosticians [4–6]. Databases containing sequences of informative loci near highly conserved regions in the genome have been developed for fungal molecular identification and phylogenetics [7, 8], and the mycological community has adopted loci such as the internal transcribed spacers (ITS) as barcodes that are used to identify fungi to a reasonable level of confidence [9]. Molecular barcodes, in general, need to be highly specific when they are used to identify pathogens. However, multiple copies and sequence homology arising from convergent or parallel evolution of genomic regions make it difficult to create robust phylogenies using a single genomic region [10–12]. When one gene, or locus, is insufficient for taxonomic differentiation, multi-locus sequence typing (MLST) has been used by plant disease diagnosticians for pathogen identification and by taxonomists for the development of phylogenetic trees [13, 14]. To provide the resolution required for species-level classification of fungi and to quantify the genetic distances among groups of fungi, additional markers such as the largest and second-largest subunits of RNA polymerase II, translation elongation factor 1-alpha, and beta-tubulin coding genes have been used [12].

With the advent of high-throughput sequencing, whole genome sequences (WGS) are now readily generated, and genes may be bioinformatically extracted for fungal identification and the creation of detailed phylogenies. Using a large set of single-copy gene orthologs extracted from high-quality reference genomes, researchers have resolved phylogenetic conflicts with genome-level comparisons [15–21]. A middle ground between WGS and the sequencing of a few discriminatory markers selected for MLST would allow for more efficient use of high throughput sequencing and provide sufficient data for fungal taxonomy and molecular fungal pathogen identification. Considering costs and bioinformatic challenges, generating manageable datasets using reduced representation sequencing has effectively provided discriminatory taxonomic power in molecular phylogenetics [22–30] that require sequence from genes of interest across large numbers of samples. Target enrichment is a technique that focuses sequencing resources on genomic regions of interest and improves its cost-effectiveness [30]. It has been adopted for elucidating phylogeny at different taxonomic levels, including lichen-forming fungi, oomycete, flowering plants, targeting a subset of genes providing phylogenetic characters [22–29]. Target enrichment has also shown its potential in breeding projects of wheat, potato, and loblolly pine through enrichment and identification of resistance genes or phenology-related genes [31–33].

While different criteria have been used to select target genes in other organisms, genes in the curated Benchmarking Universal Single-Copy Orthologs (BUSCO) datasets [34] have been particularly interesting and successfully used to resolve phylogenetic relationships within fungi and oomycetes [16, 17, 35]. Biotinylated probes have also been used to enrich loci that are of particular interest to specific branches of the tree of life, and the resulting sequences have been used to create detailed phylogenies [29, 36–39]. A method utilizing both loci previously used for fungal phylogenetics and known BUSCO genes would provide a bridge between older MLST-based methods and the complex and costly WGS approaches.

While focusing on sets of genes allows for increased taxonomic differentiation relative to using a single locus [16, 17, 35, 40], another approach to improving differentiation among fungal taxa is to increase the length of the sequences used for comparisons. This has been accomplished using probe-based enrichment and various tiling strategies to ensure short-read technologies provide sequence across complete genes of interest [27, 41–44]. Oxford Nanopore Technologies (ONT) sequencing has been used to generate long reads often surpassing 10 kbp [45], which means that just a few reads or even one read can cover the entire coding region with average lengths of 1.3 ~ 1.9 kb [46]. We hypothesized that pairing biotinylated probes designed to bind to the orthologous sequences within genes of interest with ONT long-read sequencing would reduce the number of probes required to produce sequences of entire genes of interest. Here, we describe methods to create a fungal identification platform that combines target enrichment and Nanopore sequencing. We provide data to demonstrate that enrichment dramatically increases the depth of coverage of targeted genes, and long-read sequencing provides sequences across genes of interest. Heatmaps of depth of coverage surrounding targeted loci are used to quantify the enrichment of targeted loci, and phylogenetic trees are used to demonstrate accurate sample classifications that may be used for species identification.

## Result

### Probe design

Targets selected from BUSCO datasets and other fungal phylogenetics studies are described in Table S1. To capture the diversity within fungi, we identified between 17,963 and 226,908 sequences from each target (Table S2) within the 386 publicly available fungal genome reference sequences downloaded from the National Center for Biotechnology Information (NCBI) Reference Sequence Database (RefSeq) [47] (Table S3). We then clustered the orthologous sequences for each target into 128 to 3360 clusters and identified the consensus 120-bp sequences within each cluster for probe design (Table S2). Probe sequences with similarity over 85% were removed, resulting in the final set of 25,735 120-mer probes (Table S4).

### *In silico* evaluation of target capturing

Three hundred eighty-six species that were included in probe design were used for *in silico* evaluation to access the number of probes that capture each phylum. As expected, all phyla were captured by our probe set. While probes were not designed using an even number of genera across the different phyla, the number of probes matching within each phylum closely reflects the number of representative genomes within each phylum (Table 1). To evaluate the efficiency of target capture across the fungal kingdom, we tested the probes using 100 species (Table S5) that became available after the probes were designed (accessed on 12/13/2022). Using the 85% matching criteria, our probe set is expected to capture targeted genes from most of the 100 genomes added to the RefSeq database following the initial design, with two exceptions coming from the Microsporidia genomes (Table 1).

**Table 1:** Summary of potential probe hybridization derived from in-silico validation. Probes were aligned with 383 fungal reference genomes available when probes were created, and an additional 100 new reference genomes available at the end of 2022

Database access date	Phylum	Number of assemblies per phylum^b^		Number of hybridized assemblies^c^	Number of aligned probes^d^	Average number of hits per phylum
July, 2021^a^	Ascomycota	295		295	618	629
	Basidiomycota	70		70	241	252
	Chytridiomycota	3		3	123	129
	Microsporidia	10		10	64	65
	Mucoromycota	4		4	208	333
	Zoopagomycota	1		1	260	334
Dec, 2022^e^	Ascomycota	76		76	652	656
	Basidiomycota	13		13	127	147
	Microsporidia	3		1	2	2
	Mucoromycota	7		7	130	239
	Zoopagomycota	1		1	80	92

^a^ Three genomes (GCF_000149205.2, GCF_000149645.2, and GCF_000143185.1) are suppressed from this analysis due to the detection of contamination.

^b^ Number of assemblies used to assess the number of hits within fungal genomes.

^c^ Number of assemblies with at least 1 hit having more than 85% sequence match between the probe and genome sequence over a 102-bp (85%) alignment.

^d^ Average number of unique probes that aligned with the assemblies.

^e^ Additional 100 genomes incorporated into fungal RefSeq database.

### Potential enrichment in other organisms

Enrichment may be particularly useful when samples contain DNA from more than one species or when other sources of contaminant DNA are present. Table 2 summarizes potential off-target hits that result from matches of probes to sequence in bacteria, mammals, nematodes, and plants. The bacterial species of the Pseudomonadota phylum has been chosen for evaluation of off-target hits due to that Pseudomonadota is the biggest phylum in Bacteria domain [48] and it contains a wide range of bacterial species that have a great impact on human health, environment, and agricultural system [49–51]. One to five probes match the genomes of 42 species of the phylum Pseudomonadota (Table S6). Using *Xanthomonas* spp. (*X. cucurbitae, X. euroxanthea, X. euvesicatoria, X. prunicola*) as examples, three probes designed to target the glyceraldehyde 3-phosphate dehydrogenase-coding gene potentially match the genomes of *Xanthomonas* spp. BLASTN (version 2.10.1) [52] searches identified 15 of the 25,735 probes which may hybridize with regions of the human genome (Table S7). These probes targeted the actin, beta-tubulin, calmodulin, and histone H3 ortholog groups in fungal genomes. We evaluated potential enrichment in five plant pathogenic nematodes, including root-knot nematodes (*Meloidogyne* spp.), cyst nematodes (*Heterodera* spp. and *Globodera* spp.), the burrowing nematode (*Radopholus similis)*, *Ditylenchus dipsaci*, and the reniform nematode *Rotylenchulus reniformis* [53]. 47 probes designed to target fungal orthologs encoding 26 S Proteasome non-ATPase regulatory subunit 14, actin, beta-tubulin, elongation factor 1, eukaryotic large ribosomal subunits, and histone H3, ribosomal protein S7 domain have positive hits on ten plant pathogenic nematodes (Table S8). Among 155 evaluated plant species, cork oak (*Quercus suber*) has the largest number of matches with 581 probes designed from 20 genes (Table S9). Positive matches were also identified in the genomes of rice (*Oryza sative*), corn (*Zea mays*), common wheat (*Triticum aestivum*), tomato (*Solanum lycopersicum*), soybean (*Glycine max*), and potato (*Solanum tuberosum*) (Table S9). The analysis of potential hybridization between probes and 1010 plant viral genomes revealed no positive matches (Table S10).

**Table 2:** Summary of probes that potentially hybridize with sequences within reference genomes of members in the kingdoms: Bacteria, Metazoa, and Viridiplantae

Kingdom	Phylum	Number of assemblies	Number of hybridized assemblies^a^	Number of aligned probes per aligned phylum	Average number of hits per aligned phylum
Bacteria	Pseudomonadota	1,808	42	2	2
Metazoa	Chordata	198	198	21	68
Metazoa	Nematoda	96	80	30	60
Viridiplantae	Chlorophyta	11	11	16	54
Viridiplantae	Rhodophyta	3	2	4	4
Viridiplantae	Streptophyta	141	140	34	91

^a^ Number of assemblies with at least 1 hit.

### Sequencing results and efficiency of enrichment

As a proof-of-concept, we extracted DNA from well-characterized fungal isolates of *Fusarium circinatum*, *Sclerotinia sclerotiorum*, and *Athelia rolfsii*, captured and enriched genes of interest for sequencing to demonstrate the platform’s utility for fungal identification. Statistics of filtered reads generated from the enriched DNA library are provided in Table 3. Median read length and read quality were similar across the three samples. Over 99% of reads aligned to the reference genomes of the samples. To find the recovered genes within each sample, we first aligned each of the 120-mer probes to the reference genomes of three fungal species. Using this probe set, we captured 116 out of 14,653 genes, 101 out of 11,130 genes, and 47 out of 8,879 genes that we annotated in the *F. circinatum* (GCA_024047395.1) [54], *S. sclerotiorum* (GCA_001857865.1) [55], and *A. rolfsii* (GCA_002940785) genomes, respectively (Tables S11-S13). Annotation of recovered genes of three fungal species is presented in Table S11-13. The depth of coverage for each target was calculated to demonstrate variations in sequencing depth among the three samples. Median depth of coverage across the genes targeted by the probes was 6,035, 8,713, and 8,016 in the *F. circinatum*, *S. sclerotiorum*, and *A. rolfsii* genomes, respectively. Targeted enrichment efficiency for each sample was calculated. The median enrichment efficiency for our samples ranged from 214 to 300. Gene size (bp), the total number of reads, base counts for on-target reads depth of coverage of each target, and enrichment estimates for each gene are presented in Tables S11-S13.

**Fig. 1:**
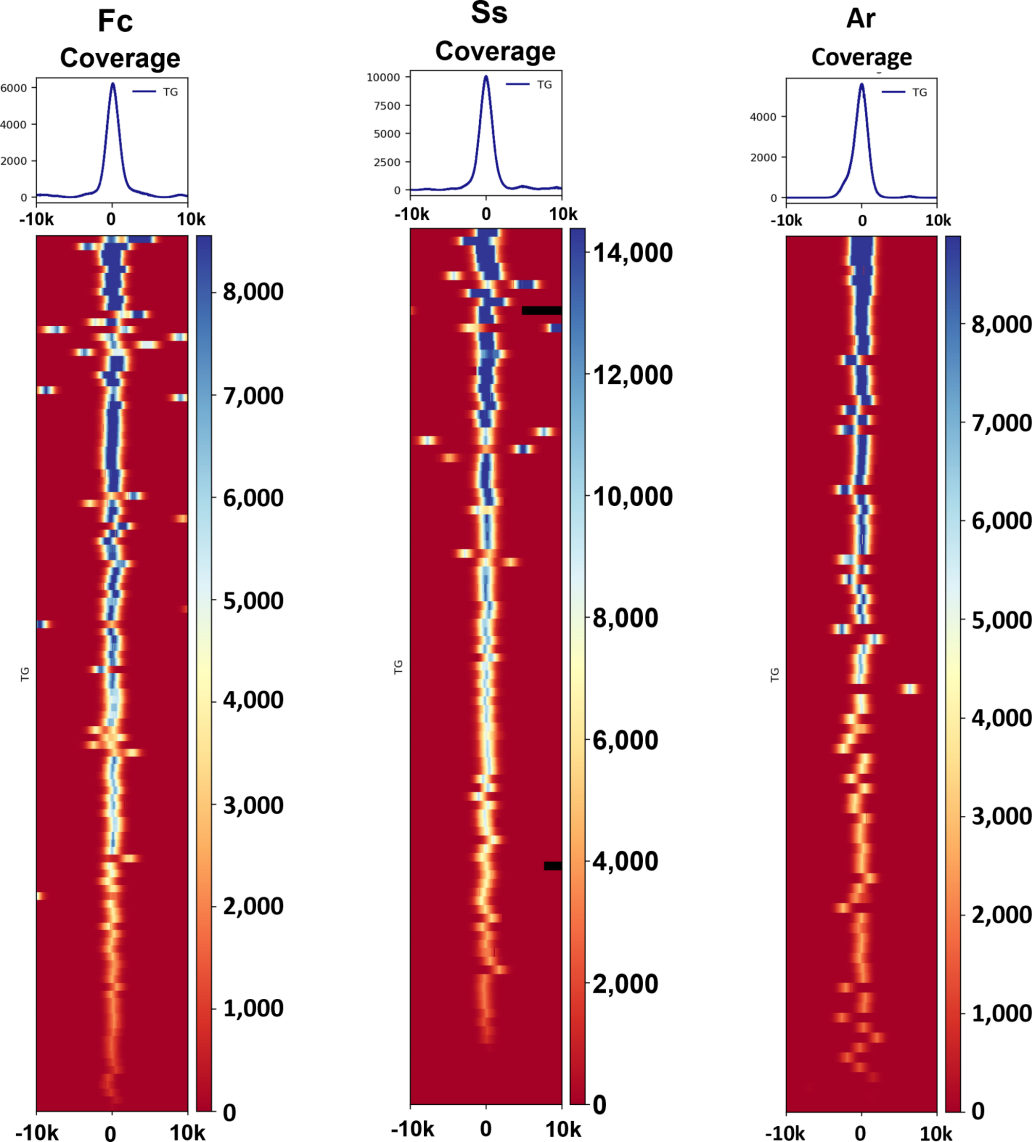
Heatmaps provide depth of coverage of sequence surrounding the targeted genes in a 20-kb window. X-axes are the flanking genome regions of each targeted gene. Color keys represent the score of depth coverage per 20-kb genome regions computed by computeMatrix. Fc: *Fusarium circinatum*; Ss: *Sclerotinia sclerotiorum*; Ar: *Athelia rolfsii*; 0 = translation starting point; TG = targeted genes

We generated heatmaps to display the depth of coverage around the translation starting point of the genes targeted by the probe set (Fig. 1). The observed depth of coverage generally follows a normal distribution with enrichment observed 2.5-kb upstream and downstream from the translation starting point (Fig. 1). While the distribution of depth of coverage is typically centered near the translation starting point, other regions of enrichment are also evident. Using *F. circinatum* as an example, some genes have two “peaks” in depth of coverage due to nearby targets (within 10-kb upstream or downstream). Downstream depth of coverage for two genes located towards the end of a chromosome in the *S. sclerotiorum* reference genome is unavailable and colored black. The location of each targeted gene is provided in Table S11-S13.

**Table 3:** Summary of sequencing results, sequence alignment, and depth of coverage for the three enriched samples. Fc: *Fusarium circinatum*; Ss: *Sclerotinia sclerotiorum*; Ar: *Athelia rolfsii*

	Fc	Ss	Ar
A. Statistics for filtered reads			
Total number of reads (n)	1,463,438	2,117,059	1,296,027
Total bases (bp)	2,427,804,453	3,329,388,951	2,104,983,341
Mean read length (bp)	1,659	1,573	1,624
Mean read quality (Phred score)	13	14	14
Median read length (bp)	1,429	1,406	1,439
Median read quality (Phred score)	14	14	14
Read length N50 (bp)	1,653	1,551	1,613
B. Statistics of reads mapped to reference genome			
Size of reference genome (bp)	46,810,763	38,906,597	32,496,039
Number of reads that align to reference genome	1,454,188	2,113,834	1,283,820
Median percent identity of alignment	95	95	90
Total bases that align to the reference genome (bp)	2,410,410,586	3,324,243,949	2,087,881,158
C. Statistics of reads mapped to targeted genes			
Number of reads that align to captured regions	997,092	1,468,619	756,085
Median percent identity of alignment	95	95	90
Total bases that cover the target genes (bp)	1,674,183,377	2,340,640,942	1,249,055,578
D. Coverage per targeted gene and enrichment efficiency			
Recovered region size (bp)	188,283	181,703	98,319
Median of depth of coverage	6,035	8,713	8,016
Median of enrichment efficiency for recovered genes	295	214	300

### Phylogenetic trees to identify closely related species

We assembled reads generated from enriched samples and present the general statistics in Table 4. We obtained 142 contigs with an N50 of 9 kb, 62 contigs with an N50 of 10 kb, *and* 80 contigs with an N50 of 9 kb from *F. circinatum*, *S. sclerotiorum*, and *A. rolfsii*, respectively. Assembly sizes are 807, 510, and 380 kb for *F. circinatum, S. sclerotiorum, and* A. *rolfsii*, respectively.

**Table 4:** Statistics of probe-enriched assemblies

Assembly Statistics	*Fusarium circinatum*	*Sclerotinia sclerotiorum*	*Athelia rolfsii*
Number of contigs	142	62	80
Largest contig (bp)	20,548	18,620	12,838
Total length (bp)	807,518	510,132	379,809
GC (%)	49	43	47
N50	8,856	9,962	8,610
L50	38	22	20

**Fig. 2:**
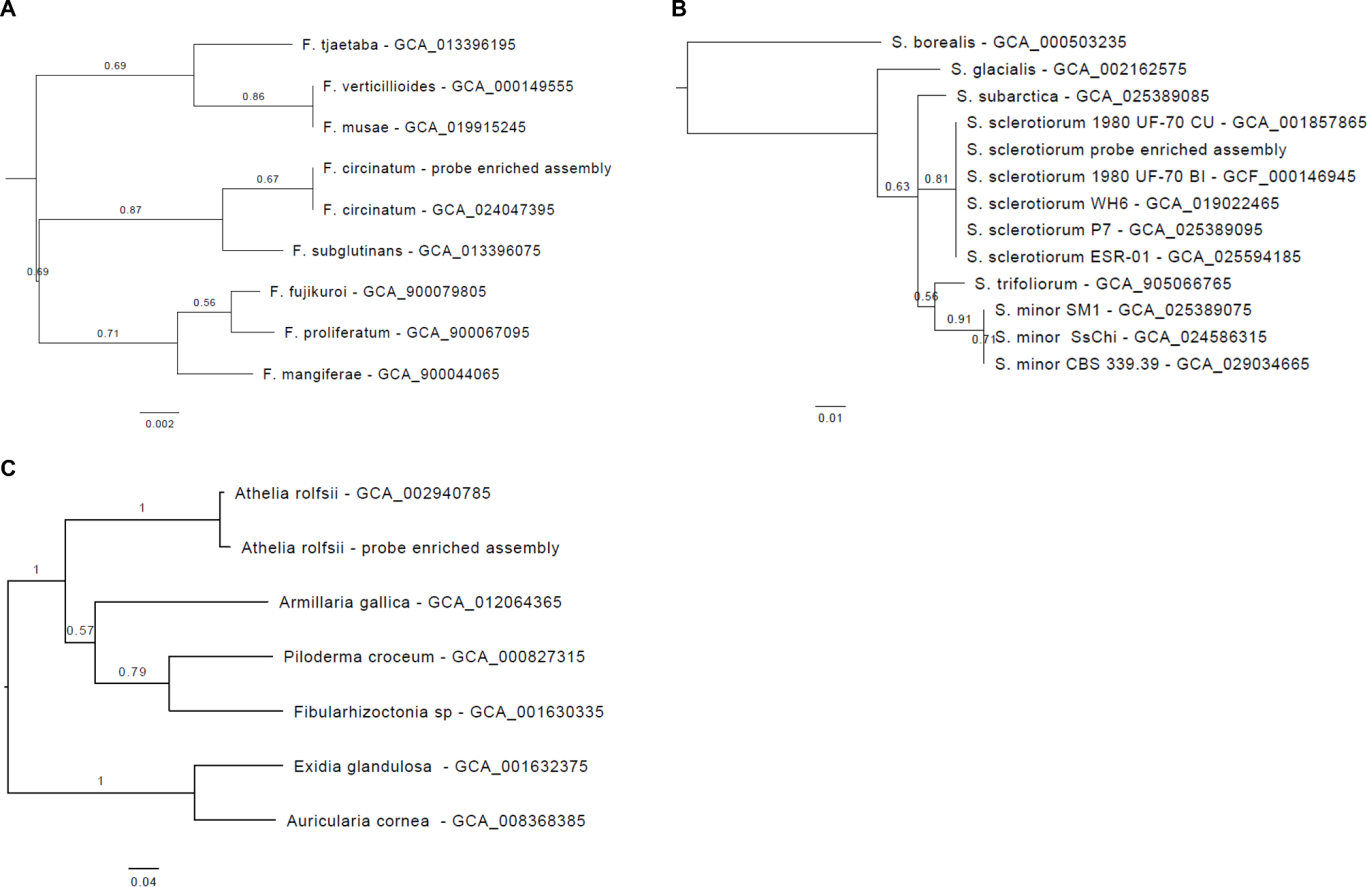
Taxonomic assignment based on majority rule consensus trees. The figures represent the phylogeny of **(A)***Fusarium* species, **(B)***Sclerotinia* species, and **(C)** species under Atheliales order. The values displayed on the branches indicate the proportion of trees analyzed that support the topology. The branch lengths are calculated based on the median of the branches across all individual trees

To assign taxonomy to these assemblies, we utilized a phylogenetic approach that involved comparing their proteomes with those of closely related assemblies available in the NCBI. The number of orthologs used to construct trees varied among samples. For *F. circinatum, S. sclerotiorum, and A. rolfsii*, we identified 269, 153, and 28 orthologs, respectively, among our assembly from enriched reads and selected reference genomes from the same genus or order for comparisons (Table S14). We generated alignments and trees for each ortholog group excluding homogenous alignments that lacked polymorphic sites. We constructed bootstrapped trees with the remaining alignments, resulting in 249, 151, and 28 trees for *Fusarium* species, *Sclerotini*a species, and species within Atheliales, respectively. We then calculated a majority rule to construct a consensus tree for each species using SumTrees from the DendroPy phylogenetic computing library (version 4.4.0) [56], which accurately placed each of the probe-enriched assemblies within the tree and provided a high degree of single-tree support for the topology (Fig. 2). For example, the support value indicates that about 166 of 249 trees (0.67) identified the same clade structure when the *F. circinatum* probe-enriched assembly was compared to its reference genome. Alignment and tree files were accessible through the Open Science Framework (OSF) [57].

## Discussion

Sequence data generated in this research following enrichment with probes designed to target a diverse set of fungal genes showed high enrichment efficiency and a high depth of coverage among target genes and provided information for high-resolution taxonomic identification. This pipeline may be used to develop protocols utilizing probe-based targeted Nanopore sequencing to identify other organisms.

### Enrichment increases the depth of coverage across targeted genes

Table 3; Fig. 1 quantify the enrichment of the genes that were targeted by our probe set. While the targets comprise an extremely small fraction of the total genome (less than 1% in the three isolates we examined), sufficient sequence data was generated to accurately classify isolates within phylogenies of related species. In the enriched samples, 60–70% of the total reads were mapped to targeted regions. For example, the enrichment of *F. circinatum* resulted in 68% of the reads coming from 116 recovered genes, a subset of the 14,563 genes in the reference genome GCA_024047395.1 [54]. As 99.4% of the reads aligned to the reference genome, off-target reads were not contaminants and were either derived from fragments not washed away near the end of the enrichment step or other regions of the genome bound by probes.

While further research and a much broader sample of fungi will be required to identify the optimal panel of probes for enriching taxonomically informative genes across the kingdom Fungi, we provide data from a diverse set of genes that provide connectivity between traditional and more recent approaches to resolve phylogenetic relationships. Historically, fungal phylogenies were developed from sequences of one or a few genes used widely in diagnostics [9–14]. More recently, BUSCO genes have been extracted from whole genome sequences to create comprehensive phylogenetic trees. To examine historically challenging relationships across the fungal kingdom, a genome-scale phylogeny of 1600 fungal species derived from 290 BUSCO was reconstructed, resolving 85% of branches of fungal phylogeny [16]. The use of large sets of genes to infer taxonomy provides clear advantages compared to the single-gene or multi-gene phylogenies that have been used to inform fungal taxonomy for decades [16, 17].

### Improvements in enrichment and taxonomic differentiation

One of the key benefits of our approach is that the enrichment not only increases the depth of coverage of targeted genes, but the long reads also recover regions flanking the targets. The capability of capturing targeted genes and regions beyond our initial targets provides a more comprehensive view of the isolate’s genomes. Although we identified some probes that may capture orthologs in organisms other than fungi, our approach provides sequences from outside the highly conserved region, providing resolution that allows for the differentiation of on- and off-target reads [23, 43]. Our results demonstrate that taxonomic assignment of probe-enriched assemblies is both possible and accurate, and this methodology also allows for further analysis by examining independent alignments and trees. Mycologists that use specific sets of genes for taxonomy may extract these genes to create MLST phylogenies that connect to previous studies.

The ability to differentiate among strains is also influenced by the number of base calling errors produced in sequencing, which may be resolved when read depth is sufficient for computational tools to correct errors. The R9.4.1 sequencing technology used in this study has an average accuracy of approximately 93–95% [58], which may not be sufficient to identify nucleotide polymorphisms and will lead to reduced resolution at the strain level. Similar to our approach (medaka), other workflows to mitigate sequencing error using computational tools for better variant calls on point mutations (SNPs) and structural variations (SV) are developed, such as algorithms that utilizing long reads [59–62] or hybrid methods using both long-read and short-read data to reduce errors [63–66]. To address the limitations of the current technology, R10.3 Nanopore, a dual-constriction biological nanopore, has been introduced, which is compatible with V14 chemistry, to offer highly accurate reads (up to 99%) comparable to Illumina sequencing [67]. Despite these challenges, recent studies have shown that Nanopore sequencing is a viable technique for species resolution [45, 68–71]. These advancements have the potential to greatly improve resolution to the race and strain level and provide even greater accuracy in future studies.

The ability to differentiate among fungal taxa improves by increasing the length of reads and providing sequences from the more variable flanking regions surrounding orthologous targets [23, 43]. We obtained reads with an average of 1.6 kb in this study, which means that few reads or even one read can cover the entire coding region of targeted genes (average length 1838 bp) as well as non-coding sequence flanking the exons. Longer reads allowed for the sequencing of whole genes, as shown in the distribution of depth of coverage around targets (Fig. 1). These long reads increase the taxonomic differentiation that can be achieved when comparing closely related fungal taxa.

These improvements may be used to increase the number of samples pooled into a single flow cell and make high throughput sequencing possible for experiments with larger numbers of samples that may be required for fungal identification, taxonomy, or diagnostic purposes. Nevertheless, the technology requires further development to reduce costs so that the approach may be used more broadly. A range of options for reducing target enrichment costs have been presented for plant systematics projects [72]. With our platform, barcoding is incorporated in the library preparation step “DNA fragmentation” [73] and our current protocol for target enrichment supports a pool of a maximum 8 indexed libraries [74]. Under the premise that pooling indexed libraries will not affect the enrichment efficiency of each library, multiplexing prior enrichment can significantly reduce per-sample costs bypassing use of ONT native barcoding. Barcoding two of the samples used in our study demonstrates the extremely high read depth for targets that resulted from the enrichment of DNA samples extracted from pure fungal cultures, further experimentation using diverse samples will provide an estimation of the maximum number of samples that may be included in a single run to produce sufficient coverage of targeted genes. To achieve satisfactory assembly completeness, a minimum of 20 to 30x long reads with 75% or more read coverage is recommended [75]. Additionally, it has been found that the number of genes reaches a plateau over 30x sequencing coverage in comparison with several other genome assemblers [76]. When infected plant tissues or environmental samples are considered, further studies will be required to estimate the maximum number of samples that can be pooled into one library to obtain sufficient read depths for targets while minimizing off-target binding from the host or other DNA sources.

## Conclusions

This study has demonstrated increases in the depth of coverage provided by enrichment and shown long read sequencing extends coverage across the genes that were targeted by the probes. Increasing the length of reads provides information from more variable sequences flanking the highly homologous regions targeted by probes, which improves taxonomic differentiation. Further experimentation is required to understand better the impacts of changes to probe design strategies to take full advantage of long-read sequencing technologies by increasing the percentage of reads coming from targeted loci. Additional research and experimentation on enriching fungal DNA from plant, animal, or environmental samples will be required to provide a cost-effective system that may be used on large numbers of samples by the mycological and diagnostics communities to consistently reconstruct fungal phylogenies and identify fungal pathogens causing disease.

## Methods

### Fungal isolates, media, and culture conditions

*S. sclerotiorum* (Lib.) de Bary (Sclerotiniaceae, Pezizomycotina) isolate UF1 was isolated from petunia in Florida, USA [77]. *A. rolfsii (*Curzi) C.C.Tu et Kimbr (Atheliaceae, Agaricomycotina) isolate 948 was kindly provided by Dr. Nicolas S. Dufault at the University of Florida. Pitch canker pathogen *F. circinatum* Nirenberg & O’Donnell (Nectriaceae, Pezizomycotina) isolate Volusia was isolated from loblolly pine [78]. For the purpose of DNA isolation, all fungal species were routinely maintained on PDA (Becton, Dickinson and Company, Franklin Lakes, NJ, USA) overlaid with a single-layer cellophane (Bio-Rad, Hercules, CA, USA), and the cultures were incubated at room temperature for seven days.

### Selection of targets

Targets were selected using two methods. Firstly, we utilized the BUSCO database, a highly curated and widely used resource for assessing genome completeness and gene content [79]. We prioritized targets that were present in the BUSCO database and have been identified as universal orthologs in fungi. Protein sequences of 17 BUSCO orthologous groups were downloaded from OrthoDB v10 [80] (Table S1). Secondly, references from the literature were used to identify genes employed as phylogenetic marker genes for fungi [81]. Protein sequences of 17 groups of phylogenetic marker genes were compiled in a previous publication [81] and were: (1) present in at least one species from each fungal phylum (or subphylum when present), (2) represented by sufficient species resolution,3) of consistent length, or 4) were of recent inclusions in the kingdom [81]. Sequences were selected from a diverse set of species that primarily had a complete, high-quality, and annotated genome in NCBI. Fungal systematic and phylogenetic consensus continues to be updated when new data becomes available. When we constructed the dataset of phylogenetic marker genes, the updated Fungal Tree of Life was used to guide the selection of genomes across the fungal kingdom [82–84]. All six “major groups of Fungi,” 10 of 12 phyla, 25 classes, and 48 orders were represented. Sequences for 17 orthologous groups of phylogenetic marker genes were downloaded from NCBI and used for probe design (Table S1). The Kyoto Encyclopedia of Genes and Genomes (KEGG) orthology (KO) of targets was obtained using BlastKOALA [85]. Sequences from both approaches were used to design a probe set that targeted 114 genes of interest (Table S1).

### Probe design

To maximize the chance of probes capturing “any” fungus, multiple representative protein sequences for each of the targeted orthologous groups were used as queries (Table S1) to search for orthologs in the 386 fungal reference genomes that were available in July/August 2021 ( Table S3) using TBLASTN (version 2.10.1) [86]. The local BLAST database created for the search was taxonomically biased across six fungal phyla: 295 Ascomycota, 70 Basidiomycota, 10 Microsporidia, 4 Mucoromycota, 3 Chytridiomycota, and 1 Zoopagomycota. The TBLASTN outputs (-outfmt 0) were parsed using a Perl script, ncbiblast_parser.pl [87], to extract the best hit for each query. Sequences were extracted from the fungal reference genomes using BEDTools (version 2.30.0) [88], then aligned sequences were clustered using CD-HIT (version 4.6.8) [89]. A Gibbs sampling motif extraction algorithm, Sequence similarities by Markov Chain Monte Carlo (SeSiMCMC; version 4.36) [90], was then used to find the consensus 120-bp sequence conserved across sequences within each cluster so that each cluster contained one or more nucleotide sequences. Clusters containing only one sequence were concatenated into a single cluster before finding the 120-bp conserved region. To remove duplicated 120-bp sequences, SeqKit (version 2.0.0) was used with rmdup option [91]. Deduplicated-FASTA files were then concatenated to create a list of 120-bp probe sequences representing all clusters of each gene. These files were submitted to Twist Bioscience Company (South San Francisco, CA, USA) for probe synthesis.

### High molecular weight genomic DNA extraction

Fungal mycelia were collected into 2-ml tubes of Lysing Matrix S (MP Biomedicals, Irvine, CA, USA) and lyophilized overnight in Labconco™ FreeZone™ 2.5 L − 50 °C Benchtop Freeze Dryers (Labconco Corporation, Kansas City, MO, USA). Freeze-dried samples were ground into fine powders using MiniG® Automated Tissue Homogenizer (SPEX SamplePrep LLC, Metuchen, NJ, USA). High molecular weight genomic DNA (HMW gDNA) was extracted from the fungal hyphae based on a modified cetyltrimethylammonium bromide (CTAB)-based method combined with a Genomic-tip 100/G (Qiagen, Hilden, Germany) to purify DNA [92]. HMW gDNA integrity, quantity and quality were accessed by Thermo Scientific™ NanoDrop™ 2000/2000c Spectrophotometers (Thermo Fisher Scientific, Waltham, MA, USA), Qubit dsDNA BR Assay Kit (Thermo Fisher Scientific), and the Agilent 2200 TapeStation system (Agilent Technologies, Santa Clara, CA, USA), respectively.

### DNA fragmentation

Before generating enriched DNA libraries for Nanopore sequencing platform, the Twist Library Kit (catalog #104,206; Twist Bioscience Company) was used for enzymatic gDNA fragmentation, and the Twist Universal Adapter System (catalog# 101,307; Twist Bioscience Company) was used according to the manufacturer’s instructions with modifications to accommodate ONT sequencing. For enzymatic fragmentation, the following reagents were mixed thoroughly by gently pipetting 4 µl of Frag/AT Buffer and 6 µl of Frag/AT Enzymes. 10 µl of this fragmentation mixture was added into the PCR 0.2-ml tube containing 40 µl of the gDNA (50 ng/µl) sample and mixed by gentle pipetting. The tube was pulse spun, placed onto pre-chilled (4 ^o^C) Applied Biosystems™ Veriti™ 96-Well Fast Thermal Cycler (hereinafter referred to as thermal cycler; Applied Biosystems, Waltham, MA, USA), and then cycling was initiated using the following steps: 20 ^o^C for 3 min; 65 ^o^C for 30 min; held at 4 ^o^C. To ligate DNA samples with Twist sequencing adapters, the user’s manual was followed. The final concentration of homogenized DNA Purification Beads is at 0.5X to retain fragments larger than 1 kb. Post-ligation purification, amplification, and post-amplification of the adapted gDNA library were performed according to the user’s manual. Quantity and size of the fragmented DNA library were assessed using the Qubit dsDNA HS Assay Kit (Thermo Fisher Scientific) and Agilent 2200 TapeStation systems before proceeding to the enrichment process.

### Enriched DNA library preparation

Twist Hybridization and Wash Kit with Amp Mix (catalog #104,178; Twist Bioscience Company), Twist Custom Panel containing our probe set (catalog #Q-142,132; Twist Bioscience Company), and Twist Blocker & Beads for Target Enrichment (catalog #100,578 and #100,983; Twist Bioscience Company) were used to generate enriched DNA libraries for Nanopore sequencing. Modifications of the original protocol were made to accommodate long-read sequencing. To reduce the reaction volume, two µg of fragmented DNA library (hereinafter referred to as DNA library) was dried using the Freeze Dryers (Labconco Corporation). The hybridization reaction consists of probes and fragmented DNA library was prepared according to the manufacturer’s instructions. Hybridization was carried out at 85 ^o^C for 16 h in the thermal cycler. Post-capture purification, amplification, and post-amplification purification of hybridized targets were conducted according to the manufacturer’s instructions. The quantity and size of the fragmented DNA library were assessed using Qubit dsDNA HS Assay Kit and Agilent 2200 TapeStation system before Nanopore sequencing.

### Nanopore sequencing and read processing

The MinION sequencer (Oxford Nanopore Technologies, Oxford, UK) was used for sequencing the enriched libraries. Sequencing followed the manufacturer’s protocols for the Ligation sequencing kit (SQK-LSK109; Oxford Nanopore Technologies). The *F. circinatum* enriched library was sequenced on a MinION flow cell (R9.4.1, Oxford Nanopore Technologies) using MinKNOW Sequencing software (version 21.05.25, Oxford Nanopore Technologies). The native barcoding kit (EXP-NBD104, Oxford Nanopore Technologies) was used to barcode and pool the *A. rolfsii* and *S. sclerotiorum* enriched DNA libraries for sequencing on another flow cell. Basecalling was conducted using Guppy (version 3.2.2) after sequencing [93]. Raw reads from the pooled samples were demultiplexed, and adaptors were trimmed using Porechop (version 0.2.4) [94]. The quality of reads was assessed using NanoPlot (version 1.30.1) [95], followed by read quality filtering via Filtlong (version 0.2.0) [96]. Reads that are longer than 1 kb and with a quality score above 7 were kept [97].

### *In silico* validation of hybridization with fungi and other species

Fungal genomes used to design baits and 100 genomes not included in the design process were used to evaluate the efficacy of the probe set (Table S5). Genome assemblies of plants, nematodes, mammals, and bacterial species of the phylum Pseudomonadota were used to quantify the possible matches or ‘off-target hits’ between probes and sequences within non-fungal genomes. Reference genome sequences from five taxonomic groups: plant viruses, bacteria, fungi, plants, and mammalian were retrieved from the NCBI RefSeq, and nematode genomes were sourced from the GenBank database [98]. BLASTN was used to analyze the number of off-target hits by searching for similarities between probe sequences and genome assemblies. The criteria for a positive hit were defined using the following probe binding metrics: 1) the percentage of identical matches ≥ 85% and 2) alignment length ≥ 102 bp or 85% of probe length, and the criteria were applied throughout this research for filtering BLASTN outputs. A description of the assemblies used for in-silico validation can be found in Tables S5-S10.

### Annotation and depth of coverage of recovered genes

The sequencing depth was calculated as follows, and detailed Python scripts were deposited on GitHub [99]. First, the reference genome is aligned to the probe sequences using BLASTN to obtain BED file storing matched positions. To annotate the captured genes, a GFF file of enriched genes containing annotation features was first extracted by comparing the BED file obtained from the previous step with genome annotation features of the reference genome. Then, the Bio.SeqIO module (interface for Biopython, version 1.76) [100] was used to extract DNA and amino acid sequences of targeted genes. Functions of recovered genes were annotated using BlastKOALA. To obtain the BAM file storing alignment between reads and targeted regions, the BED file generated from the first step was compared with the BAM file containing sequences aligned to the referent genome generated by Minimap2 (version 2.24) [101]. The statistics of BAM files were calculated by SAMtools stat (version 0.1.16) [102] and NanoPlot. Finally, the total base count and the number of reads mapping to the targeted genes were calculated with SAMtools using the bedcov command (version 0.1.17).

The depth of coverage of target genes was calculated by the ratio of total reads length (bp) to target region size (bp). The following equation was used to calculate enrichment efficiency [26]:

$$Enrichment\,efficiency=\frac{\frac{Number\,of\,reads\,that\,map\,to\,the\,target\,region}{Total\,number\,of\,reads}}{\frac{Target\,region\,size}{Haploid\,genome\,size}}$$

Heatmaps generated by deepTools (version 3.1.1) [103] were used to display depth of coverage across the 10 kb region surrounding the recovered genes.

### Phylogenetic analysis

The sequencing reads in FASTQ format were assembled using Flye (version 2.9.2-b1786) [104]. Assembled contigs were then polished with Medaka (1.7.2) to improve accuracy [105]. QUAST (version 5.0.2) was used for quality assessment of the assemblies [106]. Coding sequences of the probe-enriched assemblies were predicted using Augustus (version 3.4.0) [107]. Representative fungal genome assemblies were obtained from NCBI to facilitate comparative analysis. The single-copy ortholog core genome was determined using OrthoFinder (version 2.5.2) [108]. Alignments were performed using MAFFT (version 7.505) [109]. Finally, the phylogenetic trees of orthologs were constructed using IQ-TREE (version 2.1.0) [110], and a consensus tree was computed using SumTrees. Visualization and manipulation of phylogenetic trees were performed using FigTree (version 1.4.4) [111].

### Electronic supplementary material

Below is the link to the electronic supplementary material.

Supplementary Material 1

Supplementary Material 2

Supplementary Material 3

Supplementary Material 4

Supplementary Material 5

Supplementary Material 6

Supplementary Material 7

Supplementary Material 8

Supplementary Material 9

Supplementary Material 10

Supplementary Material 11

Supplementary Material 12

Supplementary Material 13

Supplementary Material 14

## Data Availability

Filtered reads of *F. circinatum*, *S. sclerotiorum*, and *A. rolfsii* are available through NCBI’s Sequence Read Archive (SRA) accession numbers: SRR24401655, SRR24401654, SRR24401653, respectively.

## Abbreviations

BUSCO: Benchmarking Universal Single Copy Orthologs
CTAB: Cetyltrimethylammonium bromide
HMW gDNA: High molecular weight genomic DNA
ITS: Internal transcribed spacers
KEGG: Kyoto Encyclopedia of Genes and Genomes
KO: KEGG Orthology
MLST: Multi-locus sequence typing
NCBI: National Center for Biotechnology Information
ONT: Oxford Nanopore Technology
RefSeq: National Center for Biotechnology Information Reference Sequence Database
SeSiMCMC: Sequence similarities by Markov Chain Monte Carlo
WGS: Whole genome sequences
